# Audiovisual integration of simple stimuli: spatial congruency effects unaffected by working memory load

**DOI:** 10.3389/fpsyg.2025.1594306

**Published:** 2025-07-14

**Authors:** Jingxin Chen, Qingqing Li, Hanlin Tao, Chenfei Xu, Yulin Gao, Jingjing Yang, Qiong Wu

**Affiliations:** ^1^Department of Psychology, Suzhou University of Science and Technology, Suzhou, China; ^2^College of Teacher Education, Wenzhou University, Wenzhou, China; ^3^Department of Psychology, Jilin University, Changchun, China; ^4^The School of Artificial Intelligence, Changchun University of Science and Technology, Changchun, Jilin, China; ^5^Cognitive Neuroscience Lab, Graduate School of Interdisciplinary Science and Engineering in Health Systems, Okayama University, Okayama, Japan

**Keywords:** audiovisual integration, spatial congruency, working memory load, multisensory processing, automatic integration, Bayesian analysis

## Abstract

The present study sought to investigate whether working memory (WM) load influences the spatial congruency effect in audiovisual (AV) integration using simple stimuli. Participants completed an AV localization task under three WM load conditions (0-back, 1-back, 2-back), Spatially congruent AV stimuli consistently facilitated responses regardless of working memory (WM) load. Statistical analyses found no significant interactions between WM load and audiovisual integration for reaction time (RT), accuracy, sensitivity (*d’*), or auditory enhancement effects (*p* < 0.05). Critically, Bayesian analysis in the present study provided strong evidence against the existence of such an interaction (BF ≈ 0.0001), although independent replication is warranted to confirm this finding. These findings indicate that spatially congruent AV integration is robust across different levels of working memory load, suggesting that it occurs at a low-level perceptual stage and is automatic.

## 1 Introduction

Multisensory integration (MSI) is defined as the process by which the brain combines information from different sensory modalities to form a coherent perception of the environment (Stein et al., 2010). This process is fundamental to perception and cognition, enabling individuals to interact with their surroundings more efficiently. Among various forms of MSI, audiovisual integration (AVI) plays a critical role in enhancing sensory processing and perception (Ernst and Banks, 2002; Giard and Peronnet, 1999). For instance, AVI improves reaction times, spatial precision, and perceptual judgments, supporting daily activities such as speech comprehension and navigation in dynamic environments (Spence et al., 1998; Alais and Burr, 2004; Lunn et al., 2019).

One particularly well-documented principle underlying AVI is the spatial rule, which posits that perceptual efficiency enhances significantly when visual and auditory stimuli originate from the same spatial location (Spence, 2013; Van Der Stoep et al., 2017). A wide range of behavioral studies have revealed that spatially congruent audiovisual stimuli improve localization accuracy and reaction times compared to unimodal stimuli (Li et al., 2015; Wahn and König, 2015). Moreover, event-related potential (ERP) studies indicate that spatially aligned audiovisual stimuli elicit stronger auditory N1 suppression and distinct P50 modulation within 40–60 ms post-stimulus-onset, reflecting early-stage cross-modal integration (Stekelenburg and Vroomen, 2012). Single-unit recordings from deep-layer neurons in the superior colliculus (SC) demonstrate enhanced firing rates in response to spatially congruent audiovisual stimuli, whereas incongruent inputs lead to attenuated neural activity or suppression (Meredith and Stein, 1983, 1986; Meredith et al., 1987).

Previous studies have extensively explored audiovisual integration using both simple physical stimuli (e.g., light flashes, pure tones) and complex semantic stimuli (e.g., speech-lip articulation pairs). Simple physical stimuli primarily engage bottom-up perceptual mechanisms, exhibiting automaticity and sensitivity to spatiotemporal congruence (Meredith and Stein, 1983; Teder-Sälejärvi et al., 2005; Aller et al., 2015). Conversely, semantic integration engages top-down processes requiring attentional and memory resources, and are context-dependent (Macaluso et al., 2016; Gibney et al., 2017). According to the Load Theory of Attention (Lavie et al., 2004), the integration of simple stimuli aligns with the early automatic integration hypothesis, where surplus attentional resources are passively allocated to secondary inputs. In contrast, semantic integration follows the late-stage controlled hypothesis, requiring active cognitive engagement.

Building on this distinction, a key question in multisensory research is whether audiovisual integration occurs automatically or is modulated by attentional and cognitive load (Talsma et al., 2010; Macaluso et al., 2016). Some studies propose that audiovisual integration is largely automatic and unaffected by task load (Wahn and König, 2015) or pre-attentive processing, whereas others indicate that attentional constraints can attenuate AVI effects, particularly for complex stimuli (Alsius et al., 2005, 2007; Gibney et al., 2017). The dual-task paradigm is commonly used to test this hypothesis, yielding inconsistent findings. Research demonstrates that attentional load disrupts audiovisual speech integration (e.g., the McGurk effect) but minimal influences on simple multisensory cueing effects (Alsius et al., 2005; Santangelo and Spence, 2007). Furthermore, rapid serial visual presentation (RSVP) paradigms reveal that increased attentional load attenuates and delays AVI effects (Li et al., 2020; Ren et al., 2021). However, existing studies predominantly focus on attentional load to the exclusion of working memory load, leaving unresolved whether working memory exerts comparable modulatory effects.

Attention and working memory (WM) are deeply interconnected cognitive systems that share overlapping neural substrates. Spatial attention and spatial working memory share neural mechanisms in the right-lateralized frontoparietal network, as attention is directly used to maintain spatial information in working memory (Awh and Jonides, 2001). Moreover, both spatial rehearsal and spatial selective attention show similar neural activity patterns, modulating early visual areas in similar ways both temporally and spatially (Awh and Jonides, 2001). While this anatomical overlap suggests a functional relationship (Cowan, 2010), attention and WM remain functionally dissociable processes (Fougnie, 2008). Several influential models have conceptualized this relationship. The Gate Control Theory posits that attention serves as a gating mechanism, allowing selected sensory inputs to access working memory (Chatham and Badre, 2015). The Embedded-Processes Model (Cowan et al., 2024) considers attention to be the focus of working memory—maintaining currently activated representations in conscious awareness—while working memory is defined as the temporarily activated portion of long-term memory. Meanwhile, the Multi-Component Model describes working memory as a modular system composed of a central executive and two domain-specific buffers: the phonological loop and visuospatial sketchpad (Baddeley, 2003). The central executive system coordinates multitasking by allocating attentional resources, managing processes such as the inhibition of interference and task switching, but does not itself store information. The phonological loop and visuospatial sketchpad act as domain-specific storage modules, each relying on working memory resources for specific functions (such as verbal rehearsal or image retention) (Baddeley, 2003). Notably, although these models emphasize the interdependence of attention and working memory, they also collectively support the view that the two systems are not functionally identical. Moreover, Attentional load and working memory load differ fundamentally in their influence on information processing: the former primarily affects feed forward sensory processing by enhancing the neural gain of target stimuli and suppressing competing distractor representations (Li et al., 2020). Attention is typically manipulated through perceptual or selective attention tasks, such as visual search tasks or continuous attention tasks, whereas the working memory load refers to the resources required for maintaining and manipulating information, with the N-back paradigm being a classic tool for operationalizing this construct. Furthermore, feature attention strongly modulates activity in early visual area MT (Treue and Martínez Trujillo, 1999), while WM signals are minimal or absent (Mendoza-Halliday et al., 2014), indicating the separation of these two functions at the regional level between attentional control and WM maintenance during early visual processing. Recent findings further suggest that working memory encoding and attentional modulation are dissociable, with distinct neural signatures across cortical regions (Mendoza-Halliday et al., 2024). Given this connection, our study considers that directly examining how working memory load influences the efficiency of cross-modal integration also contributes to advancing our theoretical understanding of attention mechanisms in multisensory processing. While previous studies have demonstrated that audiovisual integration can enhance working memory performance (Mastroberardino et al., 2008; Botta et al., 2011), the reverse relationship—how working memory load affects audiovisual integration—remains largely unexplored. Accordingly, whether working memory load modulates audiovisual integration-particularly within non-semantic, low-level perceptual paradigms remains an open and underexplored question. Specifically, it is unclear whether working memory load influences spatial congruence effects in audiovisual integration (AVI). Addressing this gap is crucial to understanding the extent to which cross-modal integration depends on cognitive resources.

To address this gap, the present study employed a dual-task paradigm. Participants performed an audiovisual integration (AVI) task while concurrently completing an N-back working memory task (0-back, 1-back, or 2-back) to systematically vary working memory load. Simple, non-semantic stimuli (light dots and pure tones) were used to minimize semantic influences. Spatial congruency was controlled by presenting a complex checkerboard image and a pure tone at either congruent (same side) or incongruent (opposite side) spatial locations. This congruency manipulation served as the operational measure of AVI. Spatial integration was quantified by comparing congruent audiovisual stimuli to visual-only stimuli. Additionally, to distinguish auditory alertness rather than spatial integration, we also compare incongruent audiovisual stimuli to congruent stimuli. We assume that significant differences would be observed in both comparisons.

The present study tests whether low-level spatial congruency effects persist across working memory loads. Importantly, this work extends Tang et al. (2025) in the current research topic, which demonstrated that attentional constraints modulate modality dominance during cross-modal processing. Using the Colavita paradigm, they revealed that visual or auditory expectations influence sensory dominance during later stages of audiovisual integration. While their work highlights the role of attentional modulation, our study shifts the focus to working memory load—a higher cognitive function closely related to attention. This directly addresses a key gap, as previous studies have predominantly focused on attentional load without sufficiently exploring the role of working memory in shaping early-stage multisensory integration. This work therefore complements existing cross-modal integration research while revealing novel interactions between working memory, attention, and audiovisual processing, advancing fundamental understanding of multisensory cognition.

According to the Load Theory of Attention (Lavie, 2005) and bottom-up framework of multisensory integration, task-irrelevant stimuli are automatically processed pre-attentively when cognitive resources suffice. Based on this perspective, the pre-attentive processing and automatic integration hypothesis posits that physical features of simple stimuli can be rapidly detected and integrated during early perceptual stages, a process mediated by the dorsal stream as posited in the Dual-Stream Theory of visual processing. Therefore, we hypothesize that the spatially congruent integration of simple stimuli is automated and unaffected by higher cognition working memory load. This hypothesis predicts that spatial congruency effects will manifest consistently across response times (RTs), accuracy, auditory enhancement, and sensitivity (*d’*) of AVI task, regardless of N-back task difficulty. Conversely, the Multi-Component Model of Working Memory (Baddeley, 2003), Load Theory of Cognitive Control (Lavie, 2005), and top-down integration frameworks suggest high working memory load depletes executive resources, thereby limiting available cognitive capacity for concurrent tasks. If working memory load modulates the spatial congruency effect of audiovisual integration, this modulation should emerge through measurable N-back load variations—a process potentially mediated by the ventral stream as proposed in the Dual-Stream Theory of visual processing. Such a result would be consistent with the top-down attentional modulations processing hypothesis. A corollary is the hypothesis that “WM modulates AVI”, proposing that this would manifest through interactions across RTs, accuracy, auditory enhancement, and *d’* of AVI task, predicting that multisensory integration emerges under no-load or low-load conditions but is significantly attenuated or absent under high-load conditions in N-back task.

The following experiment thus tests between the “pre-attentive processing and automatic integration” hypothesis, on the one hand, and the “top-down attentional modulations processing” and “WM modulates AVI” hypotheses, on the other. Specifically, it aims to determine whether early-stage multisensory integration operates independently of higher-level cognitive control. The experiment also examines whether low-level spatial congruency effects persist across working memory loads.

## 2 Materials and method

### 2.1 Participants

G*Power 3.1.9.2 software (Faul et al., 2009) was used to conduct an a priori power analysis for the 3 × 3 within-subjects repeated-measures ANOVA. Assuming a medium effect size (Cohen’s f = 0.25), an alpha level of 0.05, and desired power of 1 −β = 0.90, the analysis indicated that 18 participants were needed (actual power = 0.90). This medium effect size was chosen based on prior studies examining similar cognitive processing tasks, which generally report medium-sized effects for within-subject manipulations (Serdar et al., 2021). Although newer tools (e.g., simulation-based power analysis) are better suited for complex multilevel designs such as hierarchical models, G*Power remains a validated and appropriate tool for fully balanced within-subjects ANOVA designs (Brysbaert, 2019). The current study conforms to the key assumptions required for valid power estimation using G*Power, including fully crossed and categorical independent variables, a balanced design with no missing data and the application of Huynh-Feldt corrections for violations of sphericity when necessary. To ensure adequate statistical power while accounting for potential participant attrition, 33 participants were recruited. The participants were recruited from a pool of undergraduate students at Suzhou University of Science and Technology in China, with prior consent obtained from each participant. Following the ethics protocol, participants retained the unconditional right to withdraw. Two participants withdrew prematurely, and their partial data were excluded from analysis to maintain methodological consistency. Therefore, data from 31 right-handed participants (16 female) were included in the analysis, with a mean age of 20.32 years, 95% confidence interval ranging from 19.50 to 21.14. All participants reported normal or corrected-to-normal hearing and vision, with no history of neurological or psychiatric disorders. In addition, written informed consent was obtained from all participants, and the study procedures were approved in advance by the Ethics Committee of Suzhou University of Science and Technology.

### 2.2 Stimuli and apparatus

Experimental procedures were conducted in a dimly lit, electrically shielded, and sound-attenuated room located in the laboratory of Suzhou University of Science and Technology, China. Participants’ heads were stabilized using a chin rest. Visual stimuli were displayed on a 27-inch DR400 monitor (VOC, China; resolution: 1920 × 1080 pixels; refresh rate: 60 Hz) positioned 60 cm from the participant. Auditory stimuli were delivered through headphones (WH-1000XM3, Sony, Japan) with audio signals output via a Realtek Audio ALC897 sound card. To ensure accurate auditory stimulus presentation, we calibrated the output sound pressure level (SPL) using a sound level meter (AWA6228, Hangzhou Aihua, China). All stimuli were preloaded into the MATLAB/Psychtoolbox memory buffer to minimize presentation delay, ensuring synchronization between auditory and visual stimuli. MATLAB (R2022b, MathWorks, MA) with Psychtoolbox-3 was used for stimulus presentation and recorded the participants’ responses.

This experiment included three stimulus conditions: (a) unimodal visual stimuli (Only visual), (b) congruent audiovisual stimuli (AV congruent), and (c) incongruent audiovisual stimuli (AV incongruent). These stimuli were presented with equal probability within a reaction time paradigm designed to assess audiovisual integration. Congruent stimuli consisted of visual and auditory stimuli presented on the same side (left or right), whereas incongruent audiovisual stimuli were presented on opposite sides.

**FIGURE 1:**
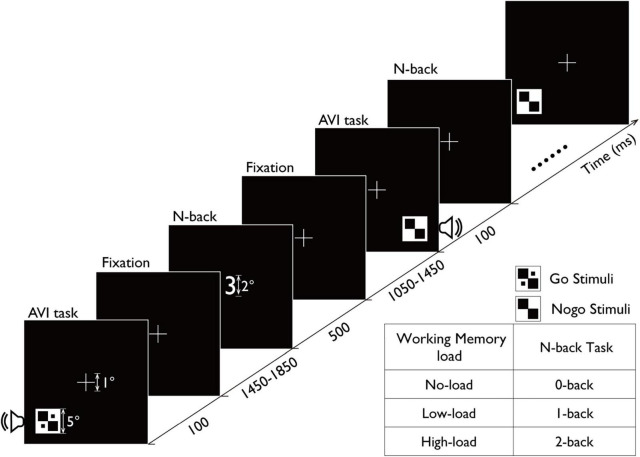
Schematic representation trial in which both the N-back task and audiovisual integration task were conducted simultaneously. Participants were required to judge the position of the target image as left or right while ignoring numbers (no-load condition), perform matching keypresses for sequentially presented numbers (low-load condition), or perform matching keypresses for numbers presented with intervals (high-load condition). In each trial, a 500-ms gaze point was presented to indicate the beginning of the new trial. A to-be-remembered number was then displayed for 500 ms, followed by a 1,050–1,450 ms N-back task response screen. Subsequently, a 100-ms audiovisual stimulus was presented, followed by a 1,450–1,850 ms audiovisual integration task response screen. Since the audiovisual integration task occurred in 80% of the trials, when this task was absent, a 3,000-ms N-back task response screen was presented after the memory item. Regarding the N-back task, participants were required to press the “2” key on the computer keyboard when a matching number appeared, as per the task requirements. For the audiovisual integration task, participants were to press the “1” key if the stimulus appeared on the left side of the screen and the “3” key if it appeared on the right side.

The visual target stimulus was a complex checkerboard image (5.2 × 5.2 cm, subtending a visual angle of 5°) with two embedded black dots. The stimulus was displayed 12° laterally (left or right) and 5° vertically below a central fixation point. Auditory stimuli in congruent and incongruent conditions consisted of 2,000 Hz pure tones presented at 60 dB SPL presented monaurally (to the left or right ear) via headphones. Visual stimuli appeared in either the lower-left or lower-right screen quadrant (at a 12° visual angle laterally and 5° below the central fixation point) against a uniform black background for improved contrast and visibility. To counteract habituation and minimize anticipatory responses, this study incorporated non-target simple checkerboard image (5.2 × 5.2 cm; 5° visual angle) requiring participants to withhold responses (Figure 1). The visual and auditory stimuli were presented with synchronous onset. All stimuli were presented for 100 ms.

The six stimulus types (three target and three non-target) are as follows. The three target stimulus types included: visual target stimuli (V+, complex checkerboard image), congruent audiovisual target pairs (V+A+, complex checkerboard image and 60 dB SPL pure tone presented on the same side), and incongruent audiovisual target pairs (V+A+*, complex checkerboard image and 60 dB SPL pure tone presented on opposite sides). The three non-target stimulus types included: visual non-target stimuli (V-, simple checkerboard image), congruent audiovisual non-target pairs (V- A-, simple checkerboard image and 60 dB SPL pure tone resented on the same side), and incongruent audiovisual non-target pairs (V-A-*, simple checkerboard image and 60 dB SPL pure tone presented on opposite sides). In total: three target types (V+, V+A+, V+A+*) and three non-target types (V-, V- A-, V-A-*).

Audiovisual (AV) integration trials accounted for 80% of the total trials (240/300 trials per working memory load condition). Within AV trials, target stimuli comprised 80% of trials (evenly distributed across congruent, incongruent, and unimodal target types; 64 trials per type), whereas non-target stimuli accounted for the remaining 20% (16 trials per non-target type). Each working memory load consisted of 300 trials divided into six counterbalanced blocks (50 trials per block) with randomized trial order to minimize fatigue and practice effects.

The stimuli for the N-back task consisted of 10 Arabic numerals (0–9). The N-back task was performed concurrently with the main task (Figure 1). Each digit, subtending a visual angle of 2.0° × 2.0°, was presented centrally for 500 ms. The digits were displayed in white font to provide high contrast with the background, optimizing participants’ visual perception.

### 2.3 Design

The experiment used a three (stimulus type: Only V, AV Congruent, AV Incongruent) × three (working memory load: No-load, Low-load, High-load) within-subjects factorial design. A dual-task paradigm was employed to test the hypothesis that spatial congruency modulates working memory load effects on AV integration. First, spatial congruency was controlled in the AV integration task. The spatially congruent and incongruent stimuli consisted of checkerboard images paired with auditory stimuli presented via single-channel headphones, with the visual and auditory stimuli either spatially matched or mismatched. Second, an adapted N-back paradigm (Kirchner, 1958) was implemented with three hierarchical load conditions: 0-back, 1-back, and 2-back. Task difficulty and working memory demands increased progressively with the *n* value.

### 2.4 Procedure

The experiment employed a dual-task paradigm with the audiovisual integration task as the primary task and the N-back task as a distractor task (Figure 1). Participants were required to complete two tasks in an interleaved manner.

Each trial began with a central fixation cross presented for 500 ms. Following the fixation, participants first saw a memory item digit for 500 ms, followed by a response window (1,050–1,450 ms) for the N-back task, which automatically terminated after a variable duration. Subsequently, audiovisual integration stimuli were presented for 100 ms, followed by the response window (1,450–1,850 ms) for the audiovisual integration task. Importantly, the N-back task was present in all trials (100%), whereas audiovisual integration stimuli were presented in 80% of trials (absent in 20%).

In the audiovisual integration task, participants were required to press a key (“left” or “right”) to indicate the position of the visual target. During the audiovisual incongruent condition, they were directed to ascertain the position of the visual stimulus while ignoring the auditory input. In the N-back task, each digit was presented with equal probability. The 0-back task, considered the no-load condition, required participants to ignore all other stimuli. The 1-back task, representing the low-load condition, required participants to identify whether the current stimulus matched the immediately preceding digit. The 2-back task, categorized as the high-load condition, required participants to identify whether the current stimulus matched the digit presented two trials earlier.

By increasing the difficulty of the distractor task, the allocation of working memory resources to audiovisual integration processing was systematically manipulated. The experiment included six blocks per load condition (50 trials per block), with each block containing 50 trials. Participants were permitted to take breaks between blocks. Additionally, each load condition was presented in separate blocks, and their order was randomized and counterbalanced across participants. Before the experiment began, all participants completed 20 practice trials per condition to confirm task understanding.

### 2.5 Data analysis

All statistical analyses were performed using two open-source software platforms: JASP (Version 0.19.3; JASP Team, 2024) for frequentist hypothesis testing, and R 4.3.2 (R Core Team, 2023) for Bayesian hypothesis testing.

### 2.6 Analysis of distractor task performance

To examine the effect of the N-back task and verify whether participants accurately performed the interference task, accuracy percentages across different working memory load conditions were analyzed.

### 2.7 Analysis of the AV integration task

Performance in the audiovisual integration task was assessed using reaction time (RT, ms), accuracy (ACC), and sensitivity estimates (*d’*) for unimodal visual stimuli, congruent audiovisual stimuli and incongruent audiovisual stimuli. Additionally, the auditory enhancement effect was computed for congruent and incongruent audiovisual stimuli.

For all participants, RTs from correct responses were included in the analysis, except for trials with RTs exceeding ± 3 standard deviations from the participant’s mean RT, which were excluded as outliers. The mean RT for each trial type was computed for each participant. Accuracy was determined based on correct responses to target stimuli and correct rejections of non-target stimuli.

The sensitivity index (*d’*), which accounts for both hit rates (i.e., correct responses to targets) and false alarm rates (i.e., incorrect responses when non-target stimuli were presented), was computed using the following formula:


(1) $$d\,\text{’}=Z_{h\,i\,t\,r\,a\,t\,e}-Z_{f\,a\,l\,s\,e\,a\,l\,a\,r\,m\,r\,a\,t\,e}$$

where Z represents the inverse of the cumulative Gaussian distribution (Haatveit et al., 2010).

The auditory enhancement effect (Sommers et al., 2005), representing the percentage change in performance for congruent AV stimuli relative to visual-only stimuli and for incongruent AV stimuli relative to visual-only stimuli, was calculated as follows:


(2) $$Auditoryenhancementeffect\left(\%\right)=\left(RT_{V}-RT_{A\,V}\right)÷RT_{V}\times 100\%$$

### 2.8 Statistical analysis

For each working memory load condition (no load, low load, high load), mean RTs, accuracy, and *d’* were computed separately for different stimulus modalities: V+ (visual-only), A+V+ (audiovisual congruent), and A-V- (audiovisual incongruent).

A repeated-measures ANOVA was conducted to examine the main effects of working memory load (three levels: no load, low load, high load) and stimulus modality (three levels: only V, AV congruent, AV incongruent-) on audiovisual integration performance. Additionally, a separate repeated-measures ANOVA was performed to analyze differences in auditory enhancement effects across working memory load conditions.

### 2.9 Bayesian repeated-measures ANOVA and interaction analysis

To complement traditional null hypothesis significance testing (NHST) and quantify evidence for or against the effects, Bayesian repeated-measures ANOVA was performed on RTs and accuracy. As principled measures of evidence strength, Bayes factors (BFs) inherently circumvent stopping rule dependencies (Rouder, 2014; Dienes, 2014). For our analyses, Cauchy (0.5) distribution (Rouder et al., 2012) was adopted to maximize sensitivity to medium effect sizes—the expected range in cognitive research. Bayes factors (BF_10_) were computed to compare models, providing a measure of how strongly the data support the alternative hypothesis (H_1_) over the null hypothesis (H_0_).

Bayesian interaction analysis was conducted to evaluate whether working memory load modulates audiovisual integration. A Bayes factor for interaction (BF_10_) was computed to determine the strength of evidence for an interaction effect between working memory load and stimulus modality. Following conventional Bayesian interpretation criteria (Kass and Raftery, 1995), BF values were categorized as follows:

BF_10_ < 0.33: Substantial evidence for no interaction (supporting H_0_).
BF_10_ between 0.33 and 3: Weak or inconclusive evidence.
BF_10_ > 3: Moderate to strong evidence for an interaction (supporting H_1_).
BF_10_ > 10: Strong evidence for an interaction.

This Bayesian approach allowed for a more robust interpretation of whether working memory load influences audiovisual integration by quantifying the relative likelihood of the hypotheses given the data, rather than relying solely on *p*-values.

## 3 Results

**FIGURE 2:**
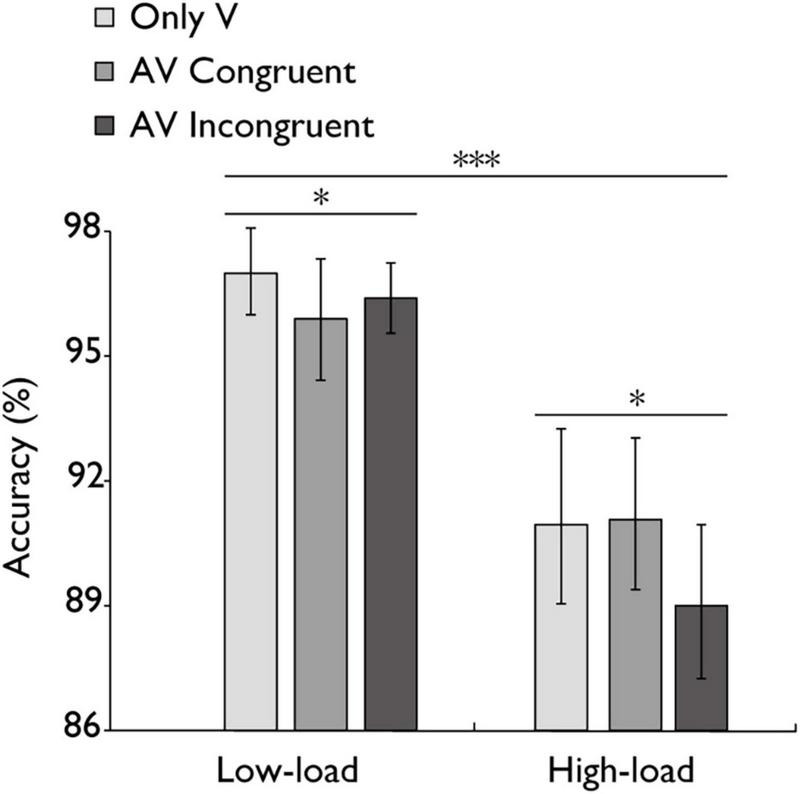
Mean accuracy (%) in the N-back task. Comparison of accuracy for the only visual (V), audiovisual congruent (AV congruent), and audiovisual incongruent (AV incongruent) conditions under low-load and high-load conditions. The horizontal lines with asterisks indicate that the main effect of working memory load is significant, and the main effect of stimulus type is significant. Error bars indicate 95% percentile bootstrap confidence intervals (1,000 resamples). ***p < 0.001, *p < 0.05.

**FIGURE 3:**
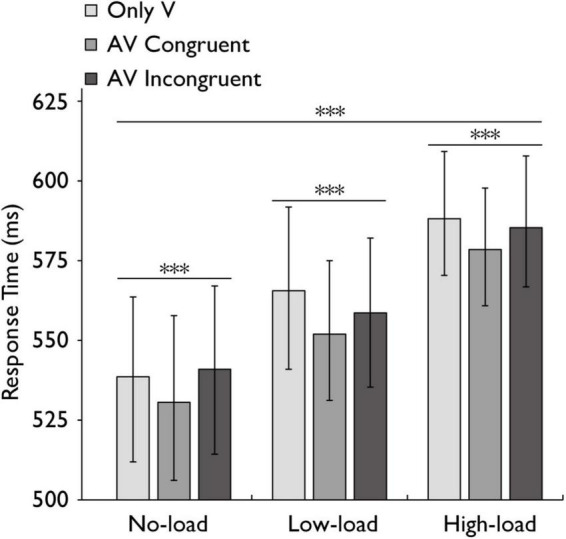
Mean reaction times in the audiovisual integration task. Comparison of mean reaction times for the only visual (V), audiovisual congruent (AV congruent), and audiovisual incongruent (AV incongruent) conditions under no-load, low-load, and high-load conditions. The horizontal lines with asterisks indicate that the main effect of working memory load is significant, and the main effect of stimulus type is significant. Error bars indicate 95% percentile bootstrap confidence intervals (1,000 resamples). ***p < 0.001.

**FIGURE 4:**
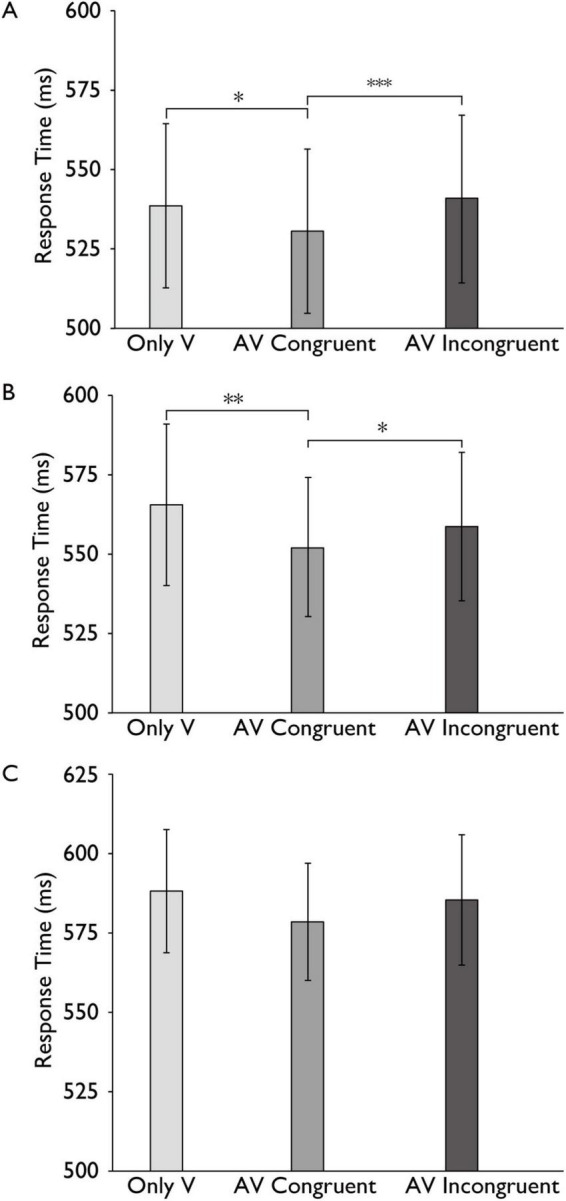
Reaction time differences in the audiovisual integration task across cognitive load conditions. Comparison of reaction times for the only visual (V), audiovisual congruent (AV Congruent), and audiovisual incongruent (AV Incongruent) conditions under no-load **(A)**, low-load **(B)**, and high-load **(C)** cognitive conditions. Horizontal brackets with asterisks indicate significant pairwise comparisons between conditions (e.g., AV Congruent vs. Only V), corrected for multiple comparisons using the Bonferroni method. Error bars represent 95% percentile bootstrap confidence intervals (1,000 resamples). ***p < 0.001, **p < 0.01, *p < 0.05.

**FIGURE 5:**
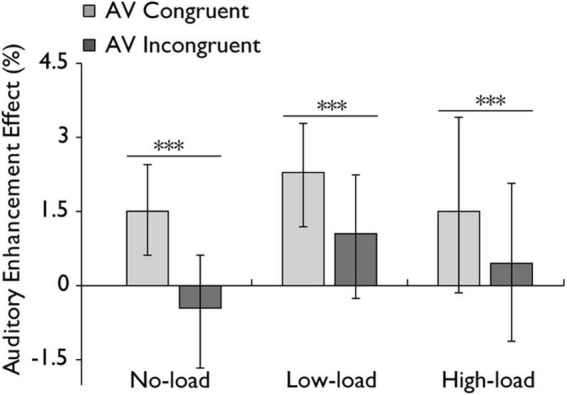
Auditory enhancement effect in the audiovisual integration task. Comparison of auditory enhancement effects for the audiovisual congruent (AV congruent) and audiovisual incongruent (AV incongruent) conditions under no-load, low-load, and high-load conditions. The horizontal lines with asterisks indicate that the main effect of stimulus type is significant. Error bars indicate 95% percentile bootstrap confidence intervals (1,000 resamples). ***p < 0.001.

**FIGURE 6:**
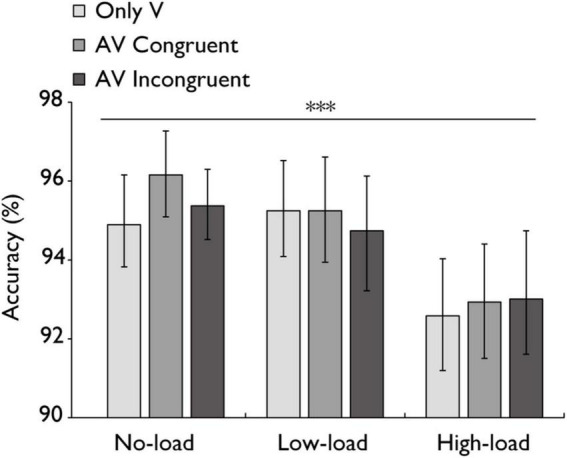
Mean accuracy (%) in the audiovisual integration task. Comparison of sensitivity accuracy (%) for the only visual (V), audiovisual congruent (AV congruent), and audiovisual incongruent (AV incongruent) conditions under no-load, low-load, and high-load conditions. The horizontal lines with asterisks indicate that the main effect of working memory load is significant. Error bars indicate 95% percentile bootstrap confidence intervals (1,000 resamples). ***p < 0.001.

**FIGURE 7:**
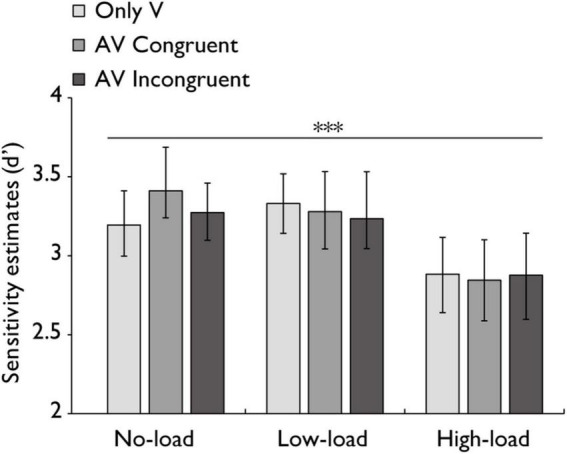
Sensitivity estimates (*d’*) in the audiovisual integration task. Comparison of sensitivity estimates (*d’*) for the only visual (V), audiovisual congruent (AV congruent), and audiovisual incongruent (AV incongruent) conditions under no-load, low-load, and high-load conditions. The horizontal lines with asterisks indicate that the main effect of working memory load is significant. Error bars indicate 95% percentile bootstrap confidence intervals (1,000 resamples). ***p < 0.001.

The findings are organized into four key sections. In Section 3.1, this study demonstrates that the N-back task successfully manipulated working memory load, as evidenced by progressively declining accuracy with increasing load (Figure 2). Section 3.2 presents reaction time analysis revealed independent main effects of memory load and stimulus type, but no significant interaction between them (Figure 3). Additionally, the planned *t*-test showed significant response facilitation for congruent (vs. incongruent) stimuli under no-load and low-load conditions, but not at high working memory load (Figure 4). Section 3.3 shows that auditory enhancement was significantly stronger for congruent versus incongruent stimuli but showed no significant interaction with working memory load (Figure 5). Section 3.4 reveals that accuracy (Figure 6) and sensitivity (Figure 7) measures exhibited significant load-dependent modulation but no significant effects of stimulus type or significant load interactions. Complete statistical outcomes are detailed below.

### 3.1 The influence of the distractor task

The mean accuracy of the N-back task across different working memory load conditions is presented in Figure 2. A repeated-measures ANOVA was conducted to assess the impact of working memory load on task accuracy. Results demonstrated a statistically highly significant main effect with a large effect of working memory load, *F*(1, 30) = 57.48, *p* < 0.001, η_*p*_^2^ = 0.657 indicating that task performance declined as memory load increased. The analysis also revealed a significant main effect of stimulus type with a medium-sized effect, *F*(1,30) = 22.325, p = 0.021, *η_*p*_*^2^ = 0.121, indicating that AV congruent trials elicited significantly better task performance than AV incongruent trials. However, the medium-sized effect of the interaction between stimulus type and working memory load was marginal, *F*(2,60) = 2.958, *p* = 0.060, *η_*p*_*^2^ = 0.090. Critically, accuracy rates exceeded 80% in all working memory load conditions, providing empirical evidence that participants maintained engagement with the distractor task rather than adopting a strategy of prioritizing the concurrent audiovisual integration task during both low-load and high-load conditions.

### 3.2 Reaction time

Following the verification of working memory load manipulation effectiveness, we examined how this factor influenced reaction times across stimulus conditions. A repeated-measures ANOVA was conducted to assess the effects of working memory load (no-load, low-load, high-load) and stimulus type (Only V, AV congruent, AV incongruent) on reaction time (RT). The analysis revealed that the main effect of working memory load was highly significant with a large effect, *F*(2,60) = 11.941, *p* < 0.001, *η_*p*_*^2^ = 0.285, while stimulus type was highly significant with a large effect, *F*(2,60) = 9.357, *p* < 0.001, *η_*p*_*^2^ = 0.238, indicating that both factors independently influenced reaction times. However, their interaction was not statistically significant with a small effect, *F*(4,120) = 0.919, *p* = 0.456, *η_*p*_*^2^ = 0.030 (Figure 3).

A Bayesian repeated-measures ANOVA was performed to examine the main effects and interaction effects of working memory load and stimulus type on RT, with participant ID as a random effect. The model including both main effects yielded a Bayes Factor of 8.66 × 10^122^ compared to the null model (random effects only). The Bayes Factors for the main effects of working memory load and stimulus type were 1.70 × 10^119^ and 3159.14, respectively. For the interaction between working memory load and stimulus type, the Bayes Factor was 0.0001 (95% CI ± 2.23%).

To further examine our main hypotheses, we then analyzed this result separately under different load conditions by conducting plan-tests and post hoc analyses with Bonferroni correction (plan-tests) conducted for multiple comparisons. Under the no-load condition, the reaction time (RT) difference between AV congruent trials and V trials was significant with a small effect, *t* (30) = 3.019, *p* = 0.010, *Cohen’s d* = 0.108, indicating faster responses in the audiovisual congruent condition. Under the low-load condition, the reaction time (RT) difference between AV congruent trials and V trials was significant with a small effect, *t* (30) = 3.949, *p* = 0.001, *Cohen’s d* = 0.205, indicating faster responses in the audiovisual congruent condition. Under the high-load condition, the difference between AV congruent and V trials was not statistically significant with a small effect, *t* (30) = 1.741, *p* = 0.276, *Cohen’s d* = 0.180. Similarly, under the no-load condition, the reaction time (RT) difference between AV congruent trials and AV incongruent trials was significant with a small effect, *t* (30) = −4.540, *p* < 0.001, *Cohen’s d* = −0.139, and under the low-load condition, the reaction time (RT) difference between AV congruent trials and AV incongruent trials was significant with a small effect, *t* (30) = −2.619, *p* = 0.041, *Cohen’s d* = −0.101, indicating faster responses in the audiovisual congruent condition. The difference between AV congruent and V trials was not statistically significant with a small effect, *t* (30) = −1.464, *p* = 0.461, *Cohen’s d* = −0.127 (Figure 4).

### 3.3 Auditory enhancement effects across working memory load

Additionally, a repeated-measures ANOVA was conducted to examine the auditory enhancement effect, with stimulus type (AV congruent, AV incongruent) and working memory load (no load, low load, high load) as within-subject factors. The analysis revealed a highly significant main effect of stimulus type with a large effect, *F*(1,30) = 22.325, *p* < 0.001, *η_*p*_*^2^ = 0.427, indicating that AV congruent stimuli elicited a greater enhancement effect compared to AV incongruent stimuli. However, the main effect of working memory load was not statistically significant with a small effect, *F*(2,60) = 1.267, *p* = 0.289, *η_*p*_*^2^ = 0.041. And the interaction between stimulus type and working memory load was not statistically significant with a small effect, *F*(2,60) = 0.613, *p* = 0.525, *η_*p*_*^2^ = 0.020 (Figure 5). In addition, a Bayesian repeated-measures ANOVA was performed on the auditory enhancement effect. The main effect of stimulus type was strongly supported by the data (BF_10_ = 13.0, ± 1.0%). The main effect of working memory load was not supported (BF_10_ = 0.32, ± 0.8%). The model including both main effects showed moderate support compared to the null model (BF_10_ = 4.72, ± 3.3%). The Bayes Factor for the interaction between stimulus type and working memory load was 0.14 ( ± 9.7%), providing evidence against the presence of an interaction effect.

### 3.4 Accuracy and sensitivity estimates (*d’*)

A repeated-measures ANOVA was conducted to examine the accuracy of different stimulus types (V, AV congruent, AV incongruent) across all working memory load conditions (Figure 6). The main effect of working memory load was highly significant with a large effect, *F*(2,60) = 11.808, *p* < 0.001, *η_*p*_*^2^ = 0.282. However, the main effect of stimulus type was not statistically significant with a small effect, *F*(2,60) = 1.188, *p* = 0.312, *η_*p*_*^2^ = 0.038. The interaction between stimulus type and working memory load was also not statistically significant with a small effect, *F*(4,120) = 0.847, *p* = 0.498, *η_*p*_*^2^ = 0.027. Bayesian analysis further confirmed these results, providing strong evidence for the main effect of working memory load (BF = 27376.1), moderate evidence for the main effect of stimulus type (BF = 6.048), and strong evidence against the interaction (BF = 0.0857).

Since accuracy alone may not fully capture participants’ ability to discriminate target stimuli from non-target stimuli, sensitivity estimates (*d’*) were calculated to account for both hit rates and false alarm rates. A repeated-measures ANOVA on sensitivity estimates (*d’*) was performed with working memory load (no-load, low-load, high-load) and stimulus type (V, AV congruent, AV incongruent) as within-subjects factors. The main effect of working memory load was highly significant with a large effect, *F*(2,60) = 12.514, *p* < 0.001, *η_*p*_*^2^ = 0.294. The main effect of stimulus type was not statistically significant with a small effect, *F*(2,60) = 0.432, *p* = 0.651, *η_*p*_*^2^ = 0.014. Similarly, the interaction between factors was not statistically significant with a small effect, *F*(4,120) = 1.106, *p* = 0.357, *η_*p*_*^2^ = 0.036 (Figure 7). A Bayesian repeated-measures ANOVA was also performed on *d’* complemented the frequentist results. This analysis showed strong support for the main effect of working memory load (BF_10_ = 10,273.38, ± 0.7%) but no support for the main effect of stimulus type (BF_10_ = 0.074, ± 0.74%). The model including both main effects showed strong support compared to the null model (BF_10_ = 843.38, ± 3.01%). Finally, the interaction Bayes Factor was 0.058 ( ± 4.07%), providing strong evidence against an interaction effect.

## 4 Discussion

The current study investigated the influence of working memory load (0-back, 1-back, 2-back) on audiovisual (AV) integration and its spatial congruency effects. The results indicate that spatial congruency effects are robust and stable across conditions (see Figures 2–7). A Bayesian repeated-measures ANOVA (RM-ANOVA) further confirmed these findings, providing extreme evidence against an interaction effect between working memory load and audiovisual integration. These results support our hypothesis that spatial congruency effects in audiovisual integration are robust and resistant to cognitive resource limitations. Based on the Unity Assumption (Welch, 1999), spatial congruency allows participants to largely infer that the stimuli come from a unified source, where competition between simple stimuli is minimal. The sustained audiovisual integration under high working memory load thereby supports both the automatic integration hypothesis and pre-attentive processing mechanisms (Talsma et al., 2010), aligning with Load Theory’s (Lavie, 2005) proposition that early perceptual binding occurs automatically until reaching cognitive depletion thresholds.

This section is organized into five subsections: Section 4.1 demonstrates the robustness of spatially congruent audiovisual integration under high working memory load, supporting its reliance on early perceptual mechanisms and automatic integration processes associated with simple stimuli and bottom-up frameworks. Section 4.2 integrates Dual-Stream Theory, specifically highlighting the central role of the dorsal pathway (“Where”) in mediating spatial audiovisual tasks. Section 4.3 explores the dynamic interplay among visual dominance, alerting mechanisms, and inhibitory control under conditions of audiovisual incongruency, reflecting the engagement of the top-down attentional modulations and cognitive control processes relevant to complex stimuli. Section 4.4 demonstrates the influence of working memory load on audiovisual integration through the lens of resource competition, specifically examining the overlapping resource demands between working memory (WM) and attention (ATT). Finally, section 4.5 highlights the limited impact of working memory load imposed in the N-back paradigm on early-stage, bottom-up audiovisual integration processes.

### 4.1 The robustness of the spatial congruency effect in audiovisual integration

**FIGURE 8:**
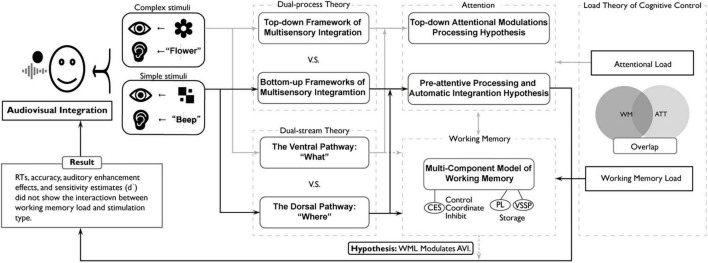
A theoretical framework for audiovisual integration under working memory load and attentional load: dissociating automatic and controlled processes. The black solid lines represent the routes supported by this study, and the gray solid lines represent the routes supported by previous studies. Moreover, the grey dashed line represents the proposed hypothesis that working memory load modulates the spatial congruency effect of audiovisual integration (WML modulates AVI)—a relationship not supported by this study. The task of this study was to examine the judgment of image positions for simple stimuli under different working memory load conditions. The results demonstrated that the audiovisual integration of simple stimuli based on spatial congruency exhibits an automatic integration process and remains dissociated from higher-level cognitive processes involving working memory load. That supports the pre-attentive processing and automatic integration hypothesis, Load Theory (Lavie, 2005) and the Dorsal Pathway Theory.

Our findings provide further evidence that spatially congruent audiovisual integration remains robust even under increased working memory load (Figures 3, 5). This result aligns with previous studies suggesting that spatial congruency facilitates multisensory integration, leading to more efficient perception (Meredith and Stein, 1983; Macaluso and Driver, 2005; Spence and Santangelo, 2009; Bolognini et al., 2010; Spence, 2013). One possible interpretation is that spatially congruent audiovisual integration occurs with minimal involvement of the central executive system, operating instead through automatic, bottom-up perceptual mechanisms. This perspective aligns with bottom-up vs. top-down frameworks of multisensory integration (see Figure 8; Talsma et al., 2010; Stein et al., 2010; O’Sullivan et al., 2021). Within these frameworks, high-level integration corresponds to top-down modulation, while low-level integration corresponds to automatic perceptual processing, such as early-stage feature binding, where sensory inputs are rapidly combined without requiring explicit cognitive control (Teder-Sälejärvi et al., 2005; Stekelenburg and Vroomen, 2012). Stein et al. (2010) emphasize that such integration forms a functional continuum, encompassing mechanisms ranging from low-level to high-level processing. The superior colliculus (SC) is mainly involved in low-level, robustly automatic multisensory integration mechanisms, whereas cortical regions (e.g., STS) mostly participate in high-level, cognitively regulated multisensory integration mechanisms (Stein et al., 2010; Talsma et al., 2010). Empirical studies substantiate the frameworks of multisensory integration. For instance, spatially congruent, simple audiovisual stimuli exhibit automatic integration during pre-attentive processing stages (Wahn and König, 2015), supporting the existence of low-level, automatic mechanisms at this end of the continuum. In contrast, semantically incongruent animal audiovisual pairs not only demonstrated no multisensory facilitation but also showed significantly attenuated interference effects under attentional load manipulations (Li et al., 2022), supporting the presence and critical role of high-level, cognitively regulated mechanisms at the opposite end. Neurophysiological evidence also supports this view, as neurons in the SC exhibit enhanced responses to spatially congruent audiovisual stimuli even without explicit attentional modulation (Stein and Stanford, 2008). Additionally, event-related potential (ERP) studies have reported that such stimuli elicit early sensory responses in the auditory and visual cortices, suggesting that integration occurs at early stages, relying minimally on working memory resources (Talsma et al., 2010). Spatially congruent audiovisual stimuli have been shown to enhance neural responses within the N1 and P2 components (approximately 100–200 ms post-stimulus), suggesting that spatial congruency facilitates the initiation of integration at low-level perceptual stages (Teder-Sälejärvi et al., 2005). However, some studies have presented findings that diverge from the bottom-up vs. top-down frameworks of multisensory integration. For instance, the crossmodal integration of emotionally salient stimuli may be rapidly mediated by limbic structures such as the amygdala, bypassing top-down regulation from the prefrontal cortex (Klasen et al., 2014). Despite its limitations in explaining task-driven dynamics, the hierarchical model provides valuable theoretical insights.

In contrast, high-level audiovisual integration, which involves cross-modal decision-making and semantic associations, has been linked to top-down regulation from the prefrontal cortex (Alsius et al., 2005; Li et al., 2020). Particularly in complex or cognitively demanding contexts, multisensory integration increasingly depends on cortical regions such as the PFC, reflecting a shift from automatic to controlled processing mechanisms (Stein et al., 2010). Given this distinction, our findings suggest that spatial congruency-driven audiovisual integration is primarily supported by low-level perceptual mechanisms and remains stable despite variations in cognitive load (see Figure 8). Nonetheless, while this integration appears highly automatic, higher cognitive functions may exert indirect influences by modulating attentional allocation or task engagement. Further research is needed to determine whether and to what extent such cognitive factors shape audiovisual integration under varying task demands. Under the simple perceptual conditions employed in this study, however, such influences appear minimal.

### 4.2 Dual-stream processing and the role of vision

Given that the task involves judgments based on the spatial location of images, we propose that vision is likely to drive integration via dorsal stream mechanisms (“Where” pathway in Figure 8), where spatial processing remains unaffected by WM load. According to the Dual-Stream Theory (Mishkin and Ungerleider, 1982; Milner and Goodale, 1993), the dorsal stream, which processes “where” information related to spatial location and motion, operates through the magnocellular pathway (Takahashi et al., 2013). This pathway rapidly transmits low-resolution information to support automatic processes including real-time spatial updating and obstacle avoidance (Jeannerod and Jacob, 2005). These features of the dorsal stream align well with the nature of the task, which requires quick, spatially audiovisual processing that is largely automatic and requires minimal reliance on working memory resources.

Neurophysiological evidence further supports this interpretation. The intraparietal sulcus (IPS) and area MT/V5 enhance spatial localization and motion control through coordinate transformation by integrating spatial information from multiple sensory modalities. Moreover, these regions also contribute to the allocation and modulation of spatial attention, thereby facilitating spatial integration (Klemen and Chambers, 2012). Furthermore, the V3A combines visual and auditory depth cues to refine motion-in-depth perception, showing pronounced activation during spatially congruent audiovisual conditions compared to attenuated but detectable responses during incongruent stimulation (Ogawa and Macaluso, 2013). These findings provide a neural basis for the observed robustness of the spatial congruency effect under increased cognitive load.

In contrast, the ventral stream processes “what” information such as object identity and semantic content, operating through the parvocellular pathway and involving cortical areas including the temporal lobe and prefrontal cortex (Goodale, 2014; Foster et al., 2012; Sheth and Young, 2016). This stream supports high-resolution, cognitively controlled processing and is more sensitive to working memory demands, especially when it comes to memory content at the semantic level. A similar dorsal-ventral organization has been identified in the auditory system: the dorsal auditory pathway (posterior temporal lobe to parietal cortex) processes spatial cues, while the ventral pathway (anterior temporal lobe to inferior frontal gyrus) is involved in pitch and object-based recognition (Alain et al., 2001; Rauschecker and Scott, 2009). This parallel structure supports the modular nature of multisensory integration, wherein distinct “what” and “where” systems operate independently but in coordination (Campbell, 2009).

Although our study does not exclude the role of the ventral pathway, particularly during the discrimination of go/no-go stimuli, the task may require visually spatial judgment, which primarily activates the dorsal pathway. In line with this dual-stream framework, our findings suggest that spatially congruent audiovisual integration primarily engages the dorsal stream’s fast, automatic processing pathway, allowing efficient integration even when working memory is taxed. This may explain why the congruency effect in our task remained robust across different working memory load conditions. In contrast, cross-modal integration involving semantics and other complex factors, likely mediated by ventral pathways, may be more susceptible to limitations in cognitive resources.

### 4.3 AV Incongruency: visual dominance or alerting vs. interference balance

While our findings primarily highlight the robustness of spatially congruent audiovisual integration, it is also important to consider the behavioral dynamics under incongruent conditions. In this study, the AV incongruent condition was designed to examine whether AV-congruent trials demonstrated AV facilitation or alerting effect. Due to the “visual dominance” paradigm implemented in our task design (where responses could be independently guided by visual information without requiring strong multisensory integration in AV-incongruent conditions), performance under AV-incongruent conditions was statistically similar to that under visual-only (V) conditions. Furthermore, the potential alerting effect and spatial interference effects may have counteracted each other, leading to statistically comparable performance between AV-incongruent and visual-only conditions (Li et al., 2020, 2022). This aligns with prior findings, such as those by Tang et al. (2025), which demonstrated that modality-specific expectations influence sensory dominance under similar cross-modal conditions. However, our results suggest that spatial congruency effects in audiovisual integration are more reliant on early-stage, automatic perceptual mechanisms, rather than being modulated by top-down attentional constraints. Additionally, these effects may involve limited contributions from the ventral stream. Together, these findings provide complementary insights into how different cognitive resources contribute to cross-modal integration.

### 4.4 The role of working memory load and its relationship to attentional resources

Although attention was not directly manipulated in the present study, our dual-task paradigm inherently engaged multiple attentional systems, such as focused and selective attention (Sohlberg and Mateer, 2001). For instance, target localization required focused attention, while selective attention was needed to suppress incongruent auditory inputs. However, these attentional processes were not the manipulated variables of interest in the present study. Instead, we focused on whether multisensory integration (specifically the spatial congruency effect) persists under limited cognitive resources, with working memory load as the key independent variable. Although the N-back task substantially engaged executive control processes, such as task updating and inhibitory control (Miyake et al., 2000), our findings indicated that the level of working memory load did not modulate the integration effect. This suggests that spatially congruent audiovisual integration operates independently of executive control or working memory maintenance mechanisms (see Figure 8).

Some studies suggest that working memory and attentional control may share common resources (see Figure 8; Oberauer, 2019; Fougnie and Marois, 2006; Lavie, 2005), while others challenge this shared-resource hypothesis (Souza and Oberauer, 2017). For example, Konstantinou et al. (2014) found that visual working memory load can reduce interference from flanker tasks, suggesting that increased cognitive demands may enhance selective attention. Similarly, Tang et al. (2025) demonstrated that modality-specific attentional expectations significantly modulate sensory dominance in cross-modal tasks, highlighting the role of attentional resources during late-stage multisensory integration. However, the present study found that early-stage spatially congruent audiovisual integration remains robust across varying working memory loads. In contrast, our findings suggest that early-stage spatially congruent audiovisual integration remains stable across varying working memory loads, supporting the hypothesis that early-stage integration processes rely on automatic perceptual mechanisms rather than shared cognitive resources.

The present study did not directly examine inter-individual variability in sustained attention or intra-individual fluctuations, and inter-individual differences in effects of task load and of complex working memory capacity, which could influence audiovisual integration under different load conditions. Additionally, as working memory load increases, inhibitory control may diminish, making individuals more susceptible to interference. In this experiment, participants were tasked with making judgments based on visual spatial location while ignoring auditory distractors. However, auditory distractors in incongruent conditions elicited both interference and alerting effects, which may have counterbalanced each other. According to Mindlessness Theory (Robertson et al., 1997; Warm et al., 2008; Epling et al., 2019), moderate cognitive load can enhance alerting effects, potentially explaining the negligible impact of working memory load on spatial integration. These findings suggest that spatial congruency effects in audiovisual integration are primarily driven by automatic processes, with minimal modulation by cognitive resource limitations.

### 4.5 Working memory load and its differential impact on early perceptual processing

Some studies have indicated that working memory load directly affects the efficiency of selective, sustained, and distributed attention through resource allocation and capacity limitations, playing a critical regulatory role, particularly in complex auditory environments (Rönnberg et al., 2022). However, the results of the present study revealed a different pattern. Our findings suggest that multisensory integration occurs prior to attentional modulation and operates independently of working memory encoding, storage, and processing (see Figure 8).

Several mechanisms may explain the differences observed between previous studies and our findings. First, there are differences in the types of sensory modality manipulations employed across experimental paradigms. Traditional studies on working memory load have often employed single-modality task designs, in which cognitive load is increased within a specific sensory modality—such as the visuospatial sketchpad or the phonological loop—to examine capacity limitations within a single sensory channel (Baddeley, 2003). In contrast, recent research has shown that when processing simple audiovisual stimuli with spatiotemporal congruency, the brain engages in object-based holistic encoding strategies rather than separately storing visual and auditory features (Arslan et al., 2025). ERP and behavioral data indicate that even under high cognitive load, simple and spatiotemporally congruent audiovisual integration persists, further supporting the view that such integration is a highly automatic, pre-attentive perceptual process. Moreover, the nature of resource competition between the working memory task and the primary task may also differ. The inferior parietal lobule (IPL) is directly involved in the storage, retrieval, and cross-modal transformation of phonological working memory, with its activation strength positively correlated with task load (Rauschecker and Scott, 2009). In this study, the memory task required the retention of visual digits, primarily consuming visual memory resources; replacing it with a dual-modality audiovisual memory task might lead to a different pattern of resource competition.

In addition to differences in the sensory modalities involved in the working memory task, the specific characteristics of the stimuli used in the tasks may also influence resource allocation. Given that the audiovisual integration task in the present study primarily involved spatial attention, future research could further employ a spatial working memory N-back task to investigate whether spatial working memory load modulates audiovisual integration under spatially congruent conditions.

These findings align with the goals of the research topic, “Attention Mechanisms and Cross-Modal Integration in Language and Visual Cognition,” by demonstrating that early-stage multisensory integration relies on automatic perceptual mechanisms rather than cognitive resource allocation. By showing how spatially congruent audiovisual integration persists under varying working memory loads, this study extends the understanding of multisensory processing and provides insights into how distinct stages of integration operate across different cognitive contexts. More importantly, the automatic nature of this fundamental mechanism may offer valuable insights for designing systems in high-cognitive-load scenarios. It suggests that systems utilizing spatially congruent audiovisual integration (e.g., driver assistance warnings, human-machine interface feedback) could maintain a robust effectiveness or robustness under demanding conditions, such as driving or complex interactions.

## 5 Conclusion and further work

The findings of this study demonstrate that, under the N-back paradigm, working memory load has no significant influence on spatial congruency-driven audiovisual integration, supporting the hypothesis of automatic integration.

Although the Bayesian analysis provided strong evidence against the interaction between working memory load of N-back task and stimulus type, independent replication is warranted to confirm these findings. Besides, cultural differences may modulate multisensory processing, and this influence warrants consideration. In addition, while our results support the idea that spatial congruency-driven audiovisual integration is predominantly governed by automatic low-level mechanisms, further theoretical and empirical research is needed to explore how different cognitive demands, such as increased task complexity or cross-modal decision-making requirements, might modulate integration effects. Future studies could investigate whether higher-order cognitive processes, including semantic integration and executive control, interact with the automatic processing of spatial congruency in more complex multisensory tasks. Therefore, the present findings should be interpreted within the scope of the current experimental design, and generalization beyond these conditions should await further empirical validation.

## Data Availability

The raw data supporting the conclusions of this article will be made available by the authors, without undue reservation.
